# Editorial: Metabolomics of Human Microbiome Studies: Recent Advances in Methods and Applications

**DOI:** 10.3389/fmolb.2021.800337

**Published:** 2021-11-30

**Authors:** Carola Parolin, Wentao Zhu, Jiangjiang Zhu

**Affiliations:** ^1^ Department of Pharmacy and Biotechnology, University of Bologna, Bologna, Italy; ^2^ Innovation Center of Pesticide Research, Department of Applied Chemistry, China Agriculture University, Beijing, China; ^3^ Human Nutrition Program, The Ohio State University, Columbus, OH, United States; ^4^ James Comprehensive Cancer Center, The Ohio State University, Columbus, OH, United States

**Keywords:** gut microbe, metabolomics, nutrition, maternal health, infant health, environmental toxicity

## Introduction

It is now well recognized that the gut microbial communities living in or on the human body are key determinants of human health and diseases (Fan and Pedersen, 2021). The high-throughput next-generation sequencing techniques have enabled the discovery of “who” makes up the human microbial community. Naturally, the next question is, “What are they doing there?” To answer this fundamental question, advances in high-sensitivity and high-throughput metabolomics technologies have enabled the comprehensive detection of thousands of small-molecule metabolites in microbial communities (Krautkramer et al., 2021). In this special issue of “Metabolomics of Human Microbiome Studies: Recent Advances in Methods and Applications”, we collected a series of publications that focused on the emerging applications of metabolomics to a variety of interesting research topics that related to human microbiome and their impact on human health, ranging from environmental toxicity (Zhou and Zhao), nutritional sciences to maternal and infant health (Dall’Asta et al.; Conta et al.; Sillner et al.; Marangoni et al.). Various modern analytical techniques, including mass spectrometry (MS), nuclear magnetic resonance (NMR) spectroscopy, and high-throughput 16S rRNA gene sequencing, have been applied to these studies, along with comprehensive data analysis and multi-omics integration. With a mix of review article and original research papers, we expect this special issue can provide evidence-based discoveries to many readers who are working in or plan to enter this area of research.

### Evaluating the Impact of Diet on Gut Microbes in Infants

As the colonization of human gut by microbes is a dynamic process that initiated from birth, studies of diet-gut microbe-microbial metabolism in infants can provide us a golden opportunity to elucidate the impacts of infant diet on the early-stage gut microbes, and eventually to human health during this important developmental phase. In this special issue, Sillner et al. first investigated the time-dependent feeding effects on the fecal metabolome of 42 healthy infants who were either exclusively breastfed, or received infant formula with/without supplement of *Bifidobacterial* mix, then compared the feeding type-dependent metabolic changes over a period of 24 months (Sillner et al.). They reported that despite various metabolites such as short chain fatty acids (SCFAs), amino acids and their catabolites, human milk oligosaccharides, monosaccharides, purine degradation products and plant-based metabolites from soy-based formula can be sensitively detected, the variations of fecal metabolome overtime could be attributed to many factors from amount of food, maternal diet of breastfed children, digestion status and sampling time. However, it is clear that increased fatty acids in breastfed children reflected fatty acid composition of breast milk, and human milk oligosaccharides (HMOs) were very characteristic for the fecal metabolome of breastfed infants; while the stool metabolome of formula-fed infants was dominated by Maillard reaction products such as N-deoxyfructosylleucylisoleucine. Furthermore, microbial metabolites 4-hydroxyphenyllactic and indolelactic acid were significantly increased in fecal samples of breastfed infants compared to the formula-fed infants in the first year. Meanwhile, several secondary bile acids (e.g., 7-oxoLCA, 3-dehydroCDCA, UDCA, and 7-epiCA) were detected with significantly higher concentrations in formula-fed children. Overall, this study revealed the feeding-type dependent differences of microbial metabolism, while time and individual variabilities also play major roles in shaping the gut microbial metabolome in the studied infants. Similarly, Conta et al. and coworkers performed another time-course study that focused on the interactions between diet and gut microbes (Conta et al.). Their study also reported that from breastfeeding to weaning to the introduction of solid food, the gut microbial community and microbial metabolites responded accordingly. In agreement with Sillner et al., their story supported that SCFAs and HMOs again served as metabolite biomarkers that reflecting the dietary transitions while shift of *Firmicutes* phylum occurred. Their story supported that the variations in the fecal metabolome reflected the infant’s diet transition, while the composition of the microbiota followed a more complex and still unstable behavior. This observation leads to the conclusion that during the infancy, it appears that the fecal metabolome is more representative of the diet changes than gut microbiota.

### Investigating the Vaginal Microbe-Metabolome Relationship During Pregnancy

Closely related to the infant health, maternal health of pregnant women is also an important research topic of microbial metabolomics studies. Among these maternal women, vaginal status (eubiosis vs. dysbiosis) profoundly impacts on women’s quality of life and reproductive health. Therefore, another field of application of metabolomics studies covered in this special issue is represented by the study of the vaginal environment and its peculiarities during gestation. Indeed, the instauration of a pregnancy reflects into modifications in the microbiome composition and, in turn, in the metabolome; the depiction and comprehension of such shaping can provide new insights into the pathophysiology of pregnancy and its outcome. The study from Marangoni et al. utilized quantitative ^1^H-NMR spectroscopy to investigate the dynamic changes that occur in the vaginal metabolome of Caucasian pregnant women at different gestational ages (first, second and third trimester) (Marangoni et al.). Throughout pregnancy, the vaginal microbiome becomes less diverse and converges to a lactobacilli-dominated status, characterized by a progressive reduction of biogenic amines and alcohols content, and a corresponding increase of lactate and branched amino acids level. In such study, high levels of leucine and isoleucine have been confirmed as hallmarks of a vaginal healthy condition. In addition, microbial and metabolic profiles of vaginal samples from women suffering a first-trimester miscarriage were analyzed, enabling to correlate high levels of selected metabolites (inosine, fumarate, xanthine, beanzoate, and ascorbate) to the spontaneous abortion. The same spectroscopic technique was employed by Dall’Asta et al. to investigate vaginal metabolomic profiles of Caucasian pregnant women in the first trimester, in order to correlate metabolites content (and the corresponding microbial structure) to pre-pregnancy diet and body mass index (BMI) (Dall’Asta et al.). The study underlined the strict inverse correlation between BMI and vaginal health status, with high levels of vaginal dysbiosis-related metabolites (e.g., methylamine) related to high pre-pregnancy BMI. For the first time, it has been demonstrated that a pre-pregnancy high intake of animal-sourced proteins correlates with an increased risk of vaginal dysbiosis, which is characterized by high content of biogenic amines and selected organic acids; on the other hand, high intake of carbohydrates favors a *Lactobacillus*-dominated vaginal environment and high levels of selected amino acids.

### Revealing the Impact of Pesticides on Gut Microbe and the Health Consequence in Host

Additionally, Zhou and Zhao, reviewed the health effects of pesticides exposure based on the measurements of gut microbiome and metabolomics (Zhou and Zhao). In the past few decades, a large number of pesticides have been widely used for plant protection. Pesticides may enter non-target organisms through multiple ways and bring potential health risks (Meng et al., 2020). Generally, the dysbiosis of gut microbiota often occurs simultaneously with the metabolic disorder of host. Unfortunately, most of these studies have only established a simple correlation between the pesticides and host gut microbiota and metabolic profile, the key roles of gut microbiota and its related metabolites in the host health effects induced by pesticide exposure of non-target organisms need further study. This article reviewed the studies on the toxic effects of pesticides based on the gut microbiome and metabolomics of host, and emphasized the roles of gut microbiota and its related metabolites in the host health effects induced by pesticide exposure. As pointed out by the authors, a comprehensive analysis of the changes in the gut microbiota and metabolic profile of host in the future will probably help to advance our understanding of the sophisticated mechanism of pesticide-induced toxic effects that mediated by gut microbes.

### Afterword

After getting into the articles included in this collection, it is evidential that the microbial metabolomics studies can tap into many biomedical questions, and we are only scratching the surface of this exciting area of research. Therefore, we foresee many more research progress to be reported in near future to further assist the scientific community, and to unveil the effects of environmental and dietary factors on human health mediated by human microbes and microbial metabolites. We also want to take this opportunity to thank all the contributors to this research topic, and the reviewers for their insightful comments and suggestions.
